# Author Correction: CRISPR-based transcriptional activation tool for silent genes in filamentous fungi

**DOI:** 10.1038/s41598-021-99654-6

**Published:** 2021-10-05

**Authors:** László Mózsik, Mirthe Hoekzema, Niels A. W. de Kok, Roel A. L. Bovenberg, Yvonne Nygård, Arnold J. M. Driessen

**Affiliations:** 1 Molecular Microbiology, Groningen Biomolecular Sciences and Biotechnology Institute, University of Groningen, Nijenborgh 7, 9747 AG Groningen, The Netherlands; 2 DSM Biotechnology Center, Alexander Fleminglaan 1, 2613 AX Delft, The Netherlands; 3 Synthetic Biology and Cell Engineering, Groningen Biomolecular Sciences and Biotechnology Institute, University of Groningen, Nijenborgh 7, 9747 AG Groningen, The Netherlands; 4 Division of Industrial Biotechnology, Department of Biology and Biological Engineering, Chalmers University of Technology, Kemivägen 10, 412 96 Gothenburg, Sweden; 5 Division of Forest Pathology, Department of Forest Mycology and Plant Pathology, Swedish University of Agricultural Sciences, Almas allé 5, 756 51 Uppsala, Sweden

Correction to: *Scientific Reports* 10.1038/s41598-020-80864-3, published online 13 January 2021

The Supplementary Information published with this Article contained errors.

In Note S2, the text formatting including green italics, red bold, yellow underline, purple text and blue underline was omitted.

The original Supplementary Information file is provided below.

These errors have now been corrected in the Supplementary Information file that accompanies the original Article.

## Supplementary Information

Supplementary Information.

