# Supplementary material for: Author Correction: CRISPR-based transcriptional activation tool for silent genes in filamentous fungi
Source: Sci Rep. 2021 Oct 5;11:20129. doi: 10.1038/s41598-021-99654-6 (PMC8492761; doi:10.1038/s41598-021-99654-6)
Supplement: Supplementary file 1 — Supplementary Information. [file 41598_2021_99654_MOESM1_ESM.docx]

Supplementary information

CRISPR-Based Transcriptional Activation Tool for Silent Genes in Filamentous Fungi

László Mózsik^†,1^, Mirthe Hoekzema^†,1,5^, Niels A.W. de Kok^1^, Roel A.L. Bovenberg^2,3^, Yvonne Nygård^1,2,4^ and Arnold J.M. Driessen^1,*^

Table S1: CRISPR and CRISPRa sgRNA targeting sequences used in this study.

Table S2: Macrophorin related masses identified by LC-MS in *macR*:OE.

Table S3: LC-MS metabolite analysis of macrophorin related metabolites from agar plug extracts.

Table S4: Oligonucleotide primers used for strain and vector construction.

Table S5: Modular Cloning and Gibson Assembly based vector construction.

Table S6: Fungal strains created in this study.

Table S7: Oligonucleotide primers used for qPCR analysis.

Figure S1: CRISPR/Cas9 mediated engineering of *penDE*-CP_*DsRed* and *macR*:OE fungal strains.

Figure S2: Representation of sgRNAs targeting promoter sequences of *penDE*-CP and *macR*.

Figure S3: Development of biomass, DsRed fluorescence and DsRed fluorescence in BioLector microbioreactors.

Figure S4: LC-MS UV-VIS chromatograms of hyphae extracts of *macR* overexpression strains.

Note S1: Complementary DNA sequence of *macR* (*Pc16g00410*) of *Penicillium rubens* DS68530

Note S2: DNA sequence of dCas9-VPR

**Table S1:** CRISPR and CRISPRa sgRNA targeting sequences with corresponding CRISPRater scores.

| **Name** | **Sequence (5’-3’)** | **CRISPRater score (CCtop)** |
| --- | --- | --- |
| *PenDE*-sgRNA_a | GACCATAGCATGACACTGAT | 0.54 |
| *PenDE*-sgRNA_b | GTCCCATCAGTGTCATGCTA | 0.60 |
| *PenDE*-sgRNA_c | ATTGGCCGTAGCCACCAATC | 0.76 |
| *PenDE*-sgRNA_d | ATGCTATGGTCCCAGATTGG | 0.81 |
| *PenDE*-sgRNA_e | CACTGATGGGACATCAACTG | 0.28 |
| *PenDE*-sgRNA_f | GGATGCAGCAGGGATACTCG | 0.40 |
| *MacR*-sgRNA_1 | GACCATAGCATGACACTGAT | 0.73 |
| *MacR*-sgRNA_2 | GTCCCATCAGTGTCATGCTA | 0.69 |
| *MacR*-sgRNA_3 | ATTGGCCGTAGCCACCAATC | 0.60 |
| *MacR*-sgRNA_4 | ATGCTATGGTCCCAGATTGG | 0.84 |
| *MacR*-sgRNA_5 | CACTGATGGGACATCAACTG | 0.73 |
| *MacR*-sgRNA_6 | GGATGCAGCAGGGATACTCG | 0.71 |
| *MacR*-sgRNA_7 | AAAAACCCACCTCCCCTCAG | 0.78 |
| *MacR*-sgRNA_8 | ACTCCTCCTCTGAGGGGAGG | 0.70 |
| *MacR*-sgRNA_9 | CTGCATGCGAACTCCCAATA | 0.52 |
| *MacR*-sgRNA_10 | GGAGTTCGCATGCAGAGAAG | 0.77 |
| *MacR*-sgRNA_11 | CTCGCCCGGGAGGTAATTGG | 0.84 |
| *MacR*-sgRNA_12 | TTAGCCCCCAATTACCTCCC | 0.68 |
| *MacR*-sgRNA_13 | CAACTTCTCAAACATCGTTC | 0.66 |
| *MacR*-sgRNA_14 | GTTTGAGAAGTTGGTGTTGG | 0.67 |
| *MacR*-sgRNA_15 | GCTATAACACGAAGATGACA | 0.48 |
| *MacR*-sgRNA_16 | TAATAGGCAGGAAAGATTCG | 0.79 |
| *MacR*-sgRNA_17 | TATGTTTTGAAGTATACCCG | 0.73 |
| *MacR*-sgRNA_18 | ACATCTGTAGTGCTTACCTC | 0.69 |
| *MacR*-sgRNA_19 | GGATTTTTCACGATACGGGG | 0.76 |
| *MacR*-sgRNA_20 | CCCCGTATCGTGAAAAATCC | 0.61 |
| T7-pen-loci-editing^1^ | GAACCAACATCATTAAGCAG | 0.70 |
| T7-*macR*:OE-editing^1^ | AATGTTCCACTCCTCCTCTG | 0.85 |

**Table S2:** Macrophorin related masses identified by LC-MS in *macR*:OE from SMP-agar plug extracts after 10 days of growth.

| **Nr** | **Compound** | **Formula** | **Theoretical mass m/z [M+H]^+^** | **Theoretical mass**  **m/z [M-H]^-^** | **t_R*_ (+ mode)** | **t_R*_ (- mode)** | **Selected Rt for mass measurement** | **Detected mass**  **m/z [M+H]^+^** | **Detected mass**  **m/z [M-H]^-^** | **PPM error (+ mode)** | **PPM error (- mode)** |
| --- | --- | --- | --- | --- | --- | --- | --- | --- | --- | --- | --- |
| 1 | Macrophorin A | C_22_H_32_O4 | 361.23734 | 359.22276 | 33.15 | 33.15 | 33.15 | 361.23776 | 359.22200 | **1.16** | **-2.12** |
| 2 | Macrophorin D | C_28_H_40_O_8_ | 505.27959 | 503.26502 | 33.91 | 33.91 | 33.91 | 505.27999 | 503.26441 | **0.79** | **-1.21** |
| 3 | 4-Oxomacrophorin D | C_28_H_38_O_8_ | 503.26394 | 501.24937 | 27.62/ 28.08/ 28.46/ 35.92 | 35.92 | 35.92 | 503.26469 | 501.24971 | **1.49** | **0.68** |
| 4 | DDM | C_24_H_46_O_11_ | 511.31132 | 509.29672 | 28.12 | 28.12 | 28.12 | 511.3128 | 509.29678 | **2.89** | **0.12** |

**Table S3:** LC-MS metabolite analysis of macrophorin related metabolites from fungal SMP-agar plug extracts after 10 days of growth. Data represented as ion intensity normalized to added internal standard DDM.

|  | Macrophorin A | Macrophorin D | 4’-Oxomacrophorin D |
| --- | --- | --- | --- |
| AMA18_0 | 0 | 0 | 1,436 |
| AMA18_1 | 0 | 0 | 12,470 |
| AMA18_2 | 0 | 0 | 4,473 |
| AMA18_3 | 0 | 0 | 5,843 |
| AMA18_4 | 117,925 | 117,925 | 80,451 |
| AMA18_5 | 771,392 | 77,170 | 52,530 |
| AMA18_6 | 2,646 | 0 | 1,989 |
| AMA18_7 | 3,456 | 0 | 418 |
| AMA18_8 | 0 | 827 | 1,157 |
| AMA18_9 | 1,391 | 449 | 0 |
| AMA18_10 | 0 | 0 | 3,899 |
| AMA18_11 | 800 | 0 | 1,947 |
| AMA18_12 | 0 | 0 | 2,857 |
| AMA18_13 | 0 | 0 | 5,682 |
| AMA18_14 | 0 | 636 | 1,071 |
| AMA18_15 | 0 | 811 | 3,323 |
| AMA18_16 | 0 | 0 | 5,503 |
| AMA18_17 | 0 | 764 | 1,491 |
| AMA18_18 | 956 | 0 | 806 |
| AMA18_19 | 0 | 1,321 | 0 |
| AMA18_20 | 0 | 1,691 | 3,113 |
| *MacR*:OE | 3,675,166 | 2,747,993 | 91,620 |

**Table S4:** Oligonucleotide primers used for strain and vector construction.

| **Part ID** | **Description** | **Template** | **Primer Pair Sequences (5'->3')** |
| --- | --- | --- | --- |
| A | *penDE*-CP_*DsRed-T1-SKL*-T*act* (80-800 bp flanks) | pVE2_10^2^  AddGene ID #154228 | F:TCGACACGCTTTACGAATTCCCATGG R:GATATGCCGTCTGCAGAGACTGCGATA |
| B | P*pcbC*-*ble*-T*cyc1 (*80bp flanks) | pJAK-109^3^ | F:AGACTCGGTGATGCAGCAAATAGCGACTGTTCGTTGCGGGGTCCGAACCCGCTCGGCAGCACCGGGCTCTCCCTACTATCCCTCGA TAGCAGTCGACTACATGTATCTGCATGTTGCATC R:TGACACTGATGGGACATCAACTGGGGCACCTCGAGTATCCCTGCTGCATCCGCCTAGTCTCTCCCCATGGGAATTCGTAAAGCGTGTCGATAAGCTTGCAAATTAAAGCCTTCGAGCG |
| C | P*pcbC*-*macR (*80bp flanks) | pVE2_19^2^  AddGene ID #154241 | F:GCTGCATTGGTCTGCCATTGC R:CGAACCTTGCGTCGTCGGCAGACGATGCAACTGAGCGATAACCGGGGTTTCTTGATTTGGCGGGGCTCGGATAAAGGCATTGGTGTCTAGAAAAATAATGGTGAAAACTTG |
| D | *PgpdA*-*ergA*-T*amdS* terbinafine marker (80bp flanks) | pCP1_45^4^ | F:CGCCCTGCTATCCCAACCCTGCACTTGTCCTCTTCTCTGCATGCGAACTCCCAATATGGTCACGATAAAAACCCACCTCCTACCGCTCGTACCATGGGTTGAG R:CAGTGCTTCAGTCGCCCAGATTCTCGATGGAGATTGGCCAGGTCAGCCATATATACCCTGCAATGGCAGACCAATGCAGCGAATTCGAGCTCGGAGTGGATCC |
| E1 | Cas9m4-VPR-1 | pYTK036^5^  AddGene ID #65143 | F:TGAAGACTTAATGGACAAGAAGTATTCTATCGGACTGGCCATCGGGACTAATAG R:TGAAGACTTGCAGCCACGTCGTAGTCTGAGAGC |
| E2 | Cas9m4- VPR-2 | Annealed oligos | F:TGAAGACTTCTGCCATCGTCCCTCAGAGCTTCCTCAAAGACGACTCAATTGACAATAAGGTGCTGACTCGCTCAGACAAGGCCAAGTCTTCA R:TGAAGACTTGGCCTTGTCTGAGCGAGTCAGCACCTTATTGTCAATTGAGTCGTCTTTGAGGAAGCTCTGAGGGACGATGGCAGAAGTCTTCA |
| E3 | Cas9m4- VPR-3 | pYTK036^5^  AddGene ID #65143 | F:TGAAGACTTGGCCCGGGGAAAGTCAGATAACGTGC R:TGAAGACTTATCCCCTCCGAGCTGTGAGAGG |
| E4 | Cas9m4- VPR-4 | pAG414GPD-dCas9-VPR^6^  AddGene ID #63801 | F:GCTGAAGACTTGGATAGCAGGGCTGACCCCAAGAAGAA R:TGAAGACTAAAGCTCAAAACAGAGATGTGTCGAAGATGGACAGT |
| F1 | HH-sgRNA-HDV “plug-and-play” 1 | pFC334^7^  AddGene ID #87846 | F:CGGTCTCTAGCGGCGTAAGCTCCCTAATTGGCCC R:CGGTCTCATCGGTGATGTCTGCTCAAGCGG |
| F2 | HH-sgRNA-HDV “plug-and-play” 2 | pICH41308^8^  AddGene Kit ID #1000000044 | F:GAAGACTCCCGACGAGACCCAGCTGGCACGACAGGTTTC R:GAAGACAAAAACGGAGACCACAGCTTGTCTGTAAGCGGATG |
| F3 | HH-sgRNA-HDV “plug-and-play” 3 | pFC334^7^  AddGene ID #87846 | F:TGGTCTCAGTTTTAGAGCTAGAAATAGCAAGTTAAAATAAGGCTAG R:CGGTCTCAGGAGGAGCCAAGAGCGGATTCCTCAGTCTCGTACGTCTC |
| G1 | Gibson unit 1, dCas9m4-VPR | pLM1_135 | F:GAATTCCTGCAGCCCCAGATCATCCTGTCTTCAGTCTTAACGCTGCAAGAATTCAAGCTTGGAG R:TGGGATGTTCCATGGTAGCTGTGAA |
| G2 | Gibson unit 2, p*40S* flank with *PgpdA*-*ergA*-T*amdS* | pCP1_135 | F:CAAGGTTCTTCTCGAAGTAGTTGTTCT R:CGCTCGTACCATGGGTTGAG |
| G3 | Gibson unit 3, sgRNA “plug-and-play” | pLM1_135 | F:ACAGGTGACTCTGGATGGC R:ACCTTCAATATCAACTCTTTCAGGGGGGGAGCGGCCTTAAGTCGGCAACGAGAGGTATGTCTAAAGT |
| H | T7-sgRNA-transcription template^1^ (penicillin-loci) | overlapping oligonucleotides | F:ATGTAATACGACTCACTATA**gAACCAACATCATTAAGCAG**GTTTCAGAGCTATGCTGGAAA* R:AAAAAAGCACCGACTCGGTGCCACTTTTTCAAGTTGATAACGAACTAGTCTTATTTCAACTTGCTATGCTGTTTCCAGCATAGCTCTGAAAC |
| I | T7-sgRNA- transcription template^1^ *macR*-OE | overlapping oligonucleotides | F:ATGTAATACGACTCACTATAg**AATGTTCCACTCCTCCTCTG**GTTTCAGAGCTATGCTGGAAA* R:AAAAAAGCACCGACTCGGTGCCACTTTTTCAAGTTGATAACGAACTAGTCTTATTTCAACTTGCTATGCTGTTTCCAGCATAGCTCTGAAAC |

*20bp sgRNA target sequence shown in bold, lowercase “g” indicates T7 transcription site

**Table S5:** Modular Cloning and Gibson Assembly based vector construction.

| **Created vector** | **Description** | **Cloned Part IDs or MoClo units** | **Recipient vector** |
| --- | --- | --- | --- |
| LM0_36 | dCas9m4-2xNLS-VPR | E1, E2, E3, E4 | pICH41308^8^ |
| LM1_100 | P*40S*-dCas9m4-2xNLS-VPR-T*tif35* | pZB0_21^2^, pLM0_36, pYN0_10^2^ | pICH47742^8^ |
| LM1_113 | sgRNA “plug-and-play” transcription unit (P*gdpA*-lacZ-HDV-T*trpC*) | F1, F2, F3 | pICH47761^8^ |
| LM2_135 | P*40S*-dCas9m4-2xNLS-VPR-T*tif35*, P*gpdA*-*ergA*, sgRNA “plug-and-play” transcription unit, PenFlanks, MoClo End-Linker on LVL2 MoClo backbone vector | pZB1_1^2^, pLM1_100, pCP1_45^4^, pLM1_113, pZB1_2^2^, pICH41800^8^ | pICH50505 (alternative of pAGM4673^8^) |
| pAMA18.0* | P*40S*-dCas9m4-2xNLS-VPR-T*tif35*, P*gpdA*-*ergA*-T*amdS*, sgRNA “plug-and-play” transcription unit on AMA1 backbone vector | G1, G2, G3 | pJAK-109 based linearized AMA1 vector^3^ |

*Vector constructed using Gibson Assembly

**Table S6:** Fungal strains created in this study**.**

| **Strain ID** | **Transformed DNA** | **Transformed strain** | **Transformation method** |
| --- | --- | --- | --- |
| *penDE*-CP_*DsRed* | Part ID A and B | DS68530 | PEG mediated RNP-based CRISPR-Cas9 editing by homologous recombination^1,9^  sgRNA transcribed using T7 polymerase from DNA of “Part ID: H” |
| *macR*:OE | Part ID C and D | DS68530 | PEG mediated RNP-based CRISPR-Cas9 editing by homologous recombination  sgRNA transcribed using T7 polymerase from DNA of “Part ID: I” |
| AMA18.0_*DsRed* (no-sgRNA control for *DsRed*) | pAMA18.0 | DS68530_*penDE*-CP_*DsRed* | PEG mediated vector transformation |
| AMA18.a | pAMA18.a | DS68530_*penDE*-CP_*DsRed* | PEG mediated vector transformation |
| AMA18.b | pAMA18.b | DS68530_*penDE*-CP_*DsRed* | PEG mediated vector transformation |
| AMA18.c | pAMA18.c | DS68530_*penDE*-CP_*DsRed* | PEG mediated vector transformation |
| AMA18.d | pAMA18.d | DS68530_*penDE*-CP_*DsRed* | PEG mediated vector transformation |
| AMA18.e | pAMA18.e | DS68530_*penDE*-CP_*DsRed* | PEG mediated vector transformation |
| AMA18.f | pAMA18.f | DS68530_*penDE*-CP_*DsRed* | PEG mediated vector transformation |
| AMA18.0 (no-sgRNA control for *macR*) | pAMA18.0 | DS68530 | PEG mediated vector transformation |
| AMA18.1 | pAMA18.1 | DS68530 | PEG mediated vector transformation |
| AMA18.2 | pAMA18.2 | DS68530 | PEG mediated vector transformation |
| AMA18.3 | pAMA18.3 | DS68530 | PEG mediated vector transformation |
| AMA18.4 | pAMA18.4 | DS68530 | PEG mediated vector transformation |
| AMA18.5 | pAMA18.5 | DS68530 | PEG mediated vector transformation |
| AMA18.6 | pAMA18.6 | DS68530 | PEG mediated vector transformation |
| AMA18.7 | pAMA18.7 | DS68530 | PEG mediated vector transformation |
| AMA18.8 | pAMA18.8 | DS68530 | PEG mediated vector transformation |
| AMA18.9 | pAMA18.9 | DS68530 | PEG mediated vector transformation |
| AMA18.10 | pAMA18.10 | DS68530 | PEG mediated vector transformation |
| AMA18.11 | pAMA18.11 | DS68530 | PEG mediated vector transformation |
| AMA18.12 | pAMA18.12 | DS68530 | PEG mediated vector transformation |
| AMA18.13 | pAMA18.13 | DS68530 | PEG mediated vector transformation |
| AMA18.14 | pAMA18.14 | DS68530 | PEG mediated vector transformation |
| AMA18.15 | pAMA18.15 | DS68530 | PEG mediated vector transformation |
| AMA18.16 | pAMA18.16 | DS68530 | PEG mediated vector transformation |
| AMA18.17 | pAMA18.17 | DS68530 | PEG mediated vector transformation |
| AMA18.18 | pAMA18.18 | DS68530 | PEG mediated vector transformation |
| AMA18.19 | pAMA18.19 | DS68530 | PEG mediated vector transformation |
| AMA18.20 | pAMA18.20 | DS68530 | PEG mediated vector transformation |

**Table S7:** Oligonucleotide primers used for qPCR analysis.

| **Gene** | **Sequences (5'->3')** |
| --- | --- |
| *DsRed-T1-SKL* | F:CCAAGGTGTACGTGAAGCAC R:CCTTGTAGATGAAGGAGCCGT |
| *macR* (*Pc16g00410*) | F:GACGACGCAAGGTTCGCT R:GTCCTGCGGTGATACTGGTC |
| *macA* (*Pc16g00370*) | F:CGGGTTCAAACACGTCCGTA R:CATCCAGTGCAACGCTAGGA |
| *macJ* (*Pc16g00320*) | F:TCTTGGGGAATTTGGTGGACA R:CAGACCCGATACTACCCAGC |
| *γ-actin* (*Pc20g11630*) | F:CTGGCGGTATCCACGTCACC R:AGGCCAGAATGGATCCACCG |


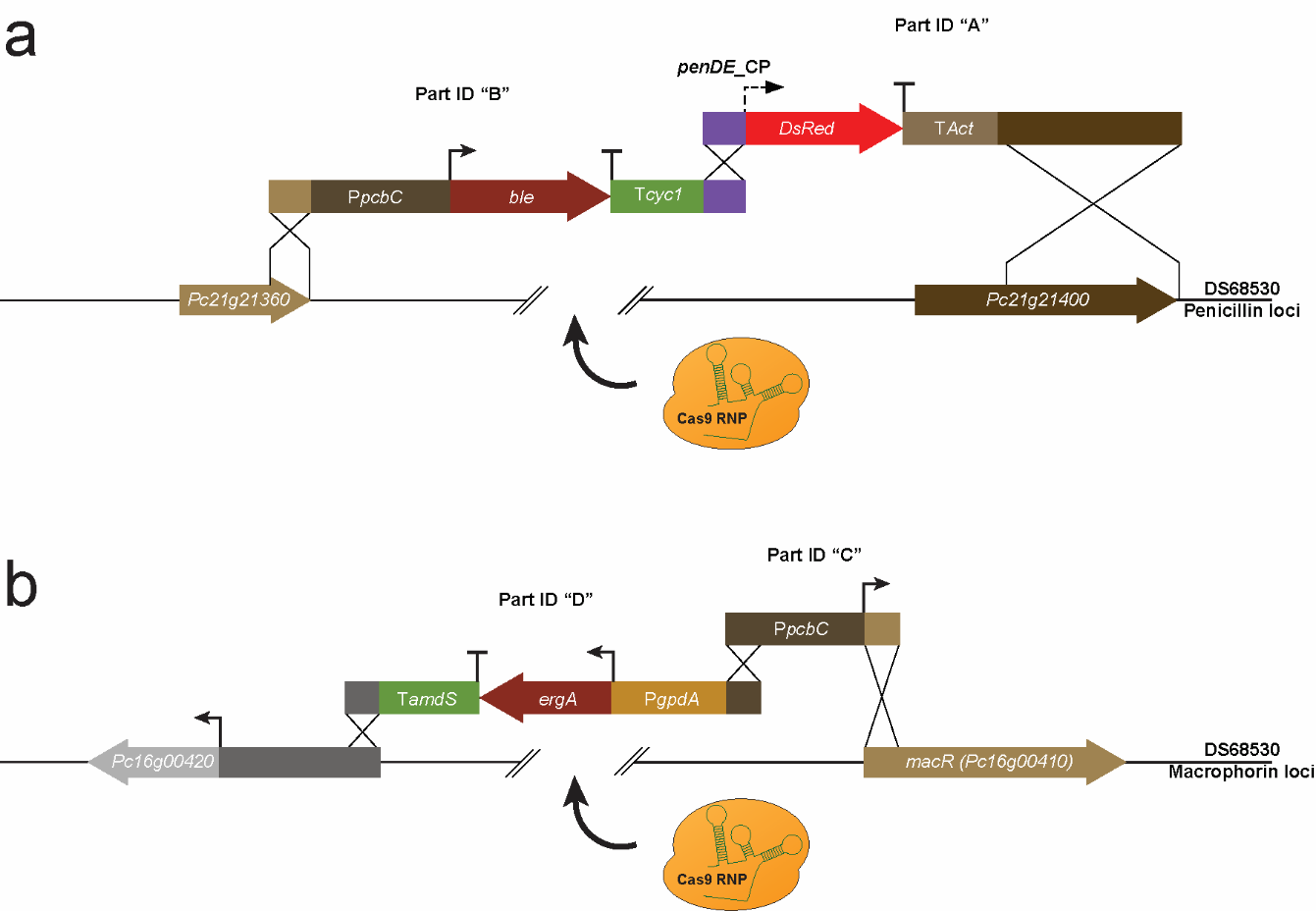


**Figure S1:** Schematic representation of CRISPR/Cas9 mediated co-transformation into DS68530 and engineering of *penDE*-CP_*DsRed* **(a)** and *macR*:OE **(b)** fungal strains, using Part ID A,B and C,D respectively. The *ergA* (terbinafine) or *ble* (phleomycin) marker provide selection and flanking regions for recombination with the marker-free cassette and the genomic DNA.

**
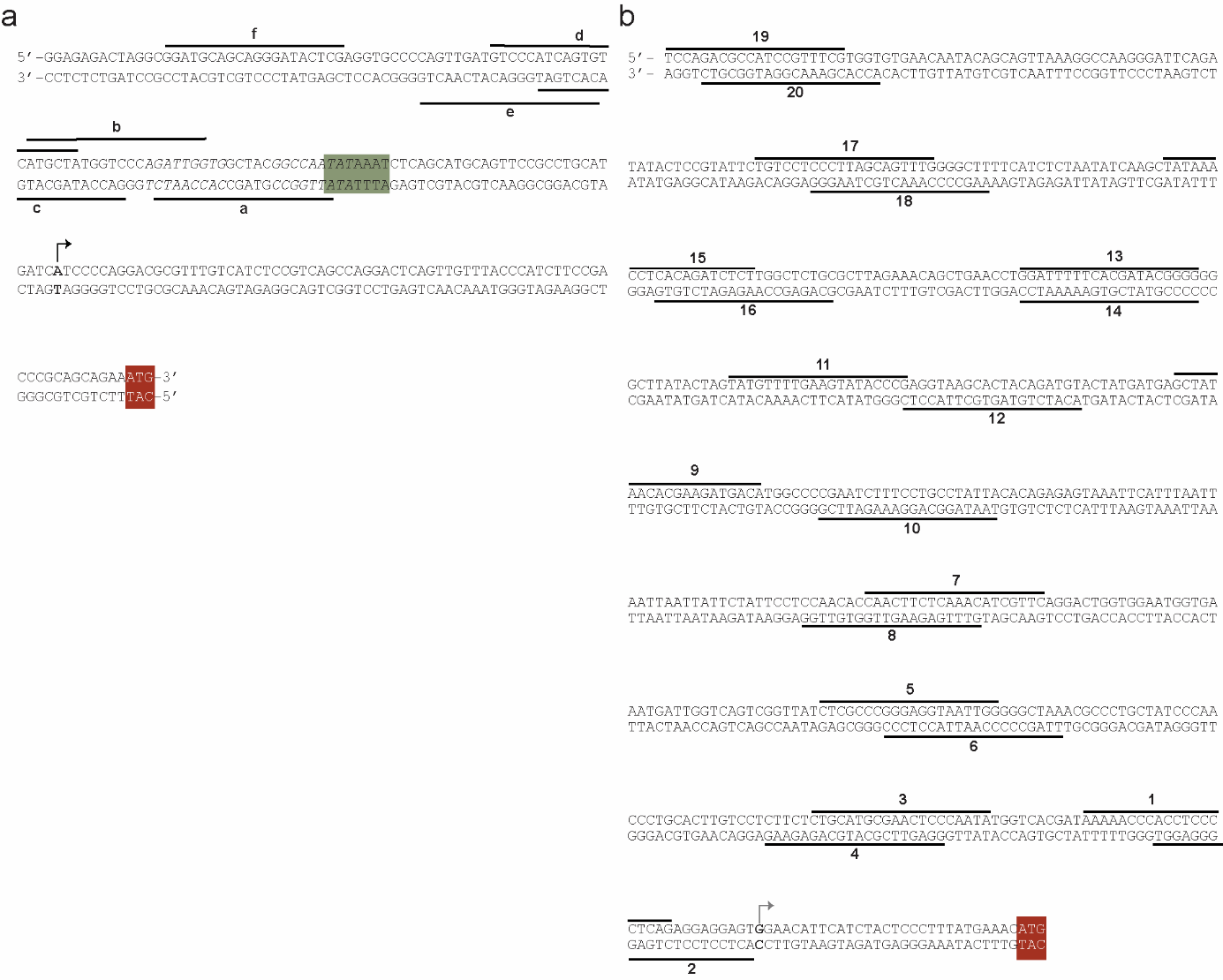
**

**Figure S2:** Representation of sgRNA targeting sequences on promoter sequences of *penDE*-CP **(a)** and *macR* **(b)**. Transcription start site (TSS) of *penDE* indicated as black arrow, predicted TSS of *macR* indicated as gray arrow. Red boxes indicates translation start codon. The green box indicates the TATA-box.

**a)**


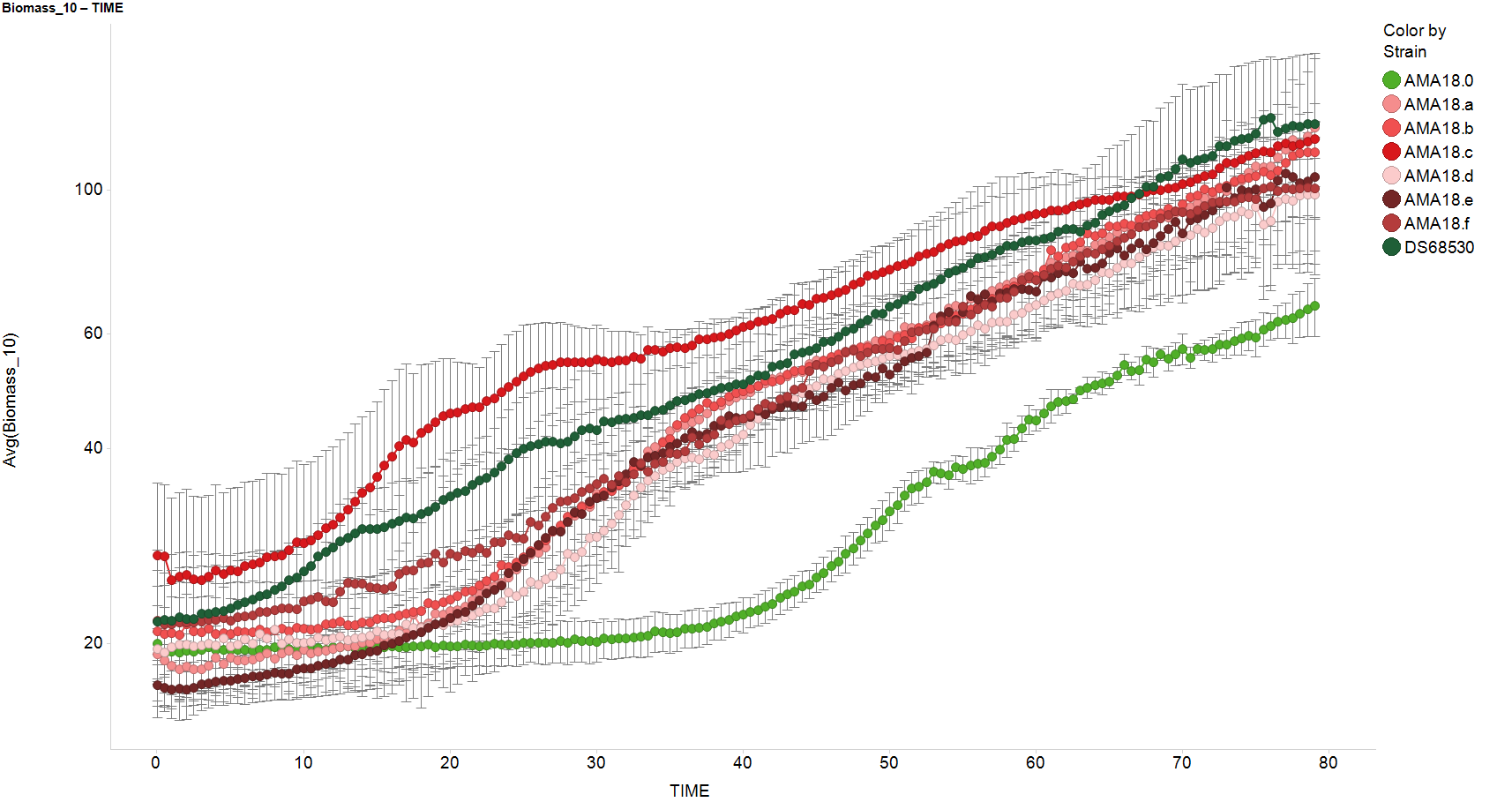


**b)**


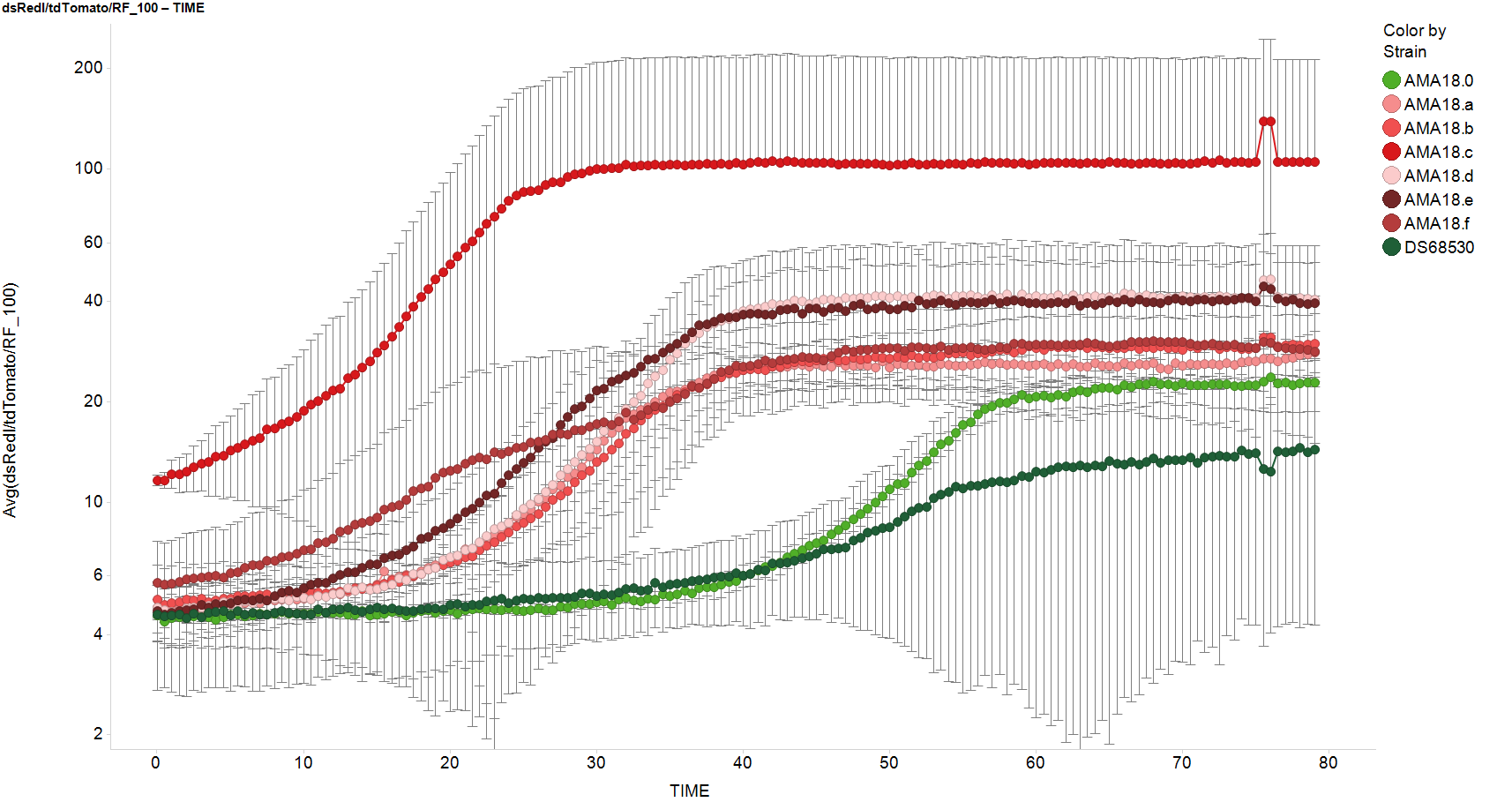


**c)**


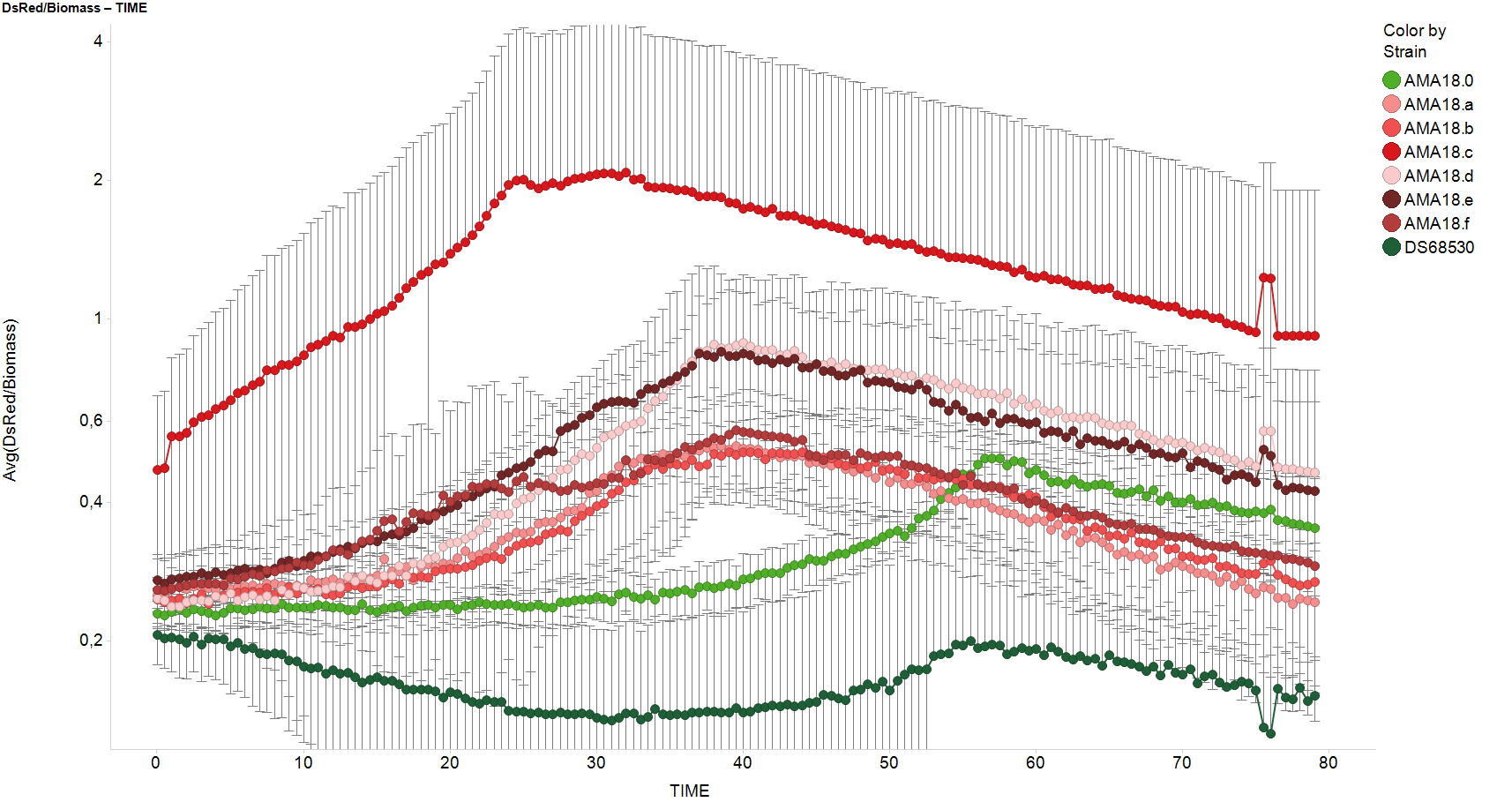


**Figure S3:** Development of **(a)** biomass **(b)** DsRed fluorescence **(c)** and DsRed fluorescence corrected for biomass for indicated CRISPRa strains in BioLector microbioreactor, compared to non-sgRNA control (AMA18.0) and *DsRed*-free parent strain (DS68530). Strains were cultivated in SMP liquid media, supplemented with terbinafine (except parent strain DS68530). Data were obtained from 3 separate experiments, each consisting of 2-3 biological replicates; error bars show the standard deviation.

**
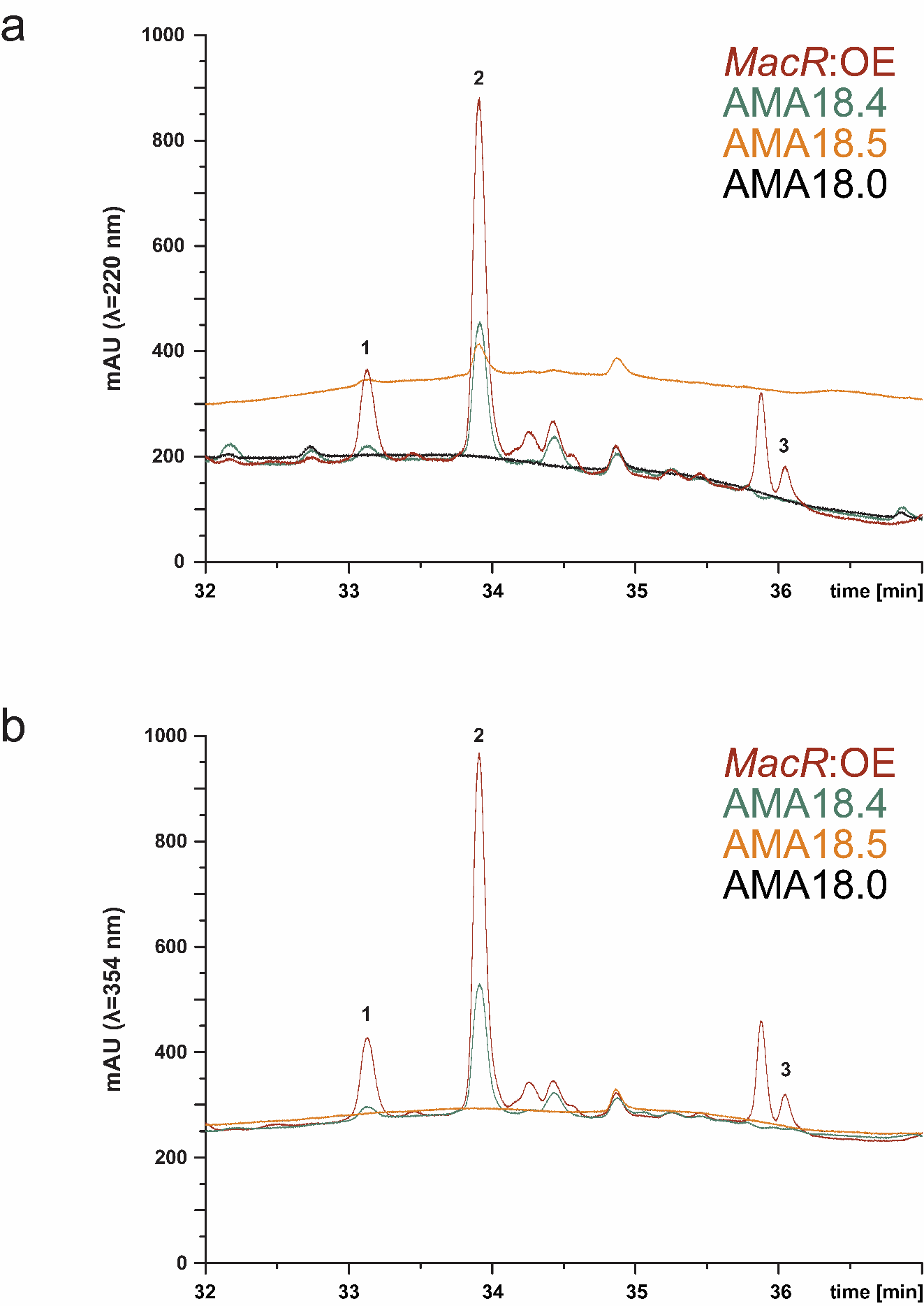
**

**Figure S4:** LC-MS UV-VIS chromatograms of hyphae extracts of CRISPRa and *macR*:OE strains analyzed at (**a)** λ=220 nm and (**b)** λ=354 nm.

**Note S1** Complementary DNA sequence of *macR* (*Pc16g00410*) of *Penicillium rubens* DS68530 as determined by Sanger sequencing.

**> *macR_*(*Pc16g00410*)_ *Penicillium*_*rubens*_DS68530_complete_cds**atgcctttatccgagccccgccaaatcaagaaaccccggttatcgctcagttgcatcgtctgccgacgacgcaaggttcgctgcggtcgcgaacagccagaatgtgccaactgtgtgcgcatgaaagagaattgtgtctaccgggccatggtccgtgacgagtccaccggccgtgtacgaccagtatcaccgcaggacaaagactccagggactctacatcggatgcccgtccagacctctcttggccgcattggggccgagacaccaatgccccataccccgataagccgtctaggcctccttctcgtcctcaagtatccgccaatgccagtccatcgccgcaacgacaagagccatacccaacagtcccttcatgggaagaggctatacaactcccaagttatcgcgatgcgaatgctcctcaaattaatggtagctcgacaactacccgcgatccctcccctgcgccatcaacaagattattccgggccccgagtgagcctccctgccgcgattatttgagtatacgacgaggtggtcgcgtgcgatacgttggtcagactttctgggggttcgttgctgggaaggaaagtctgagcgatgatttcttcgacgaaaaccgtcatgcccaccccgatctcccccttccacatatttcctcgatggggatgttcaatctcctgcggtcgctgccgaccaaacccgttagtgataccctgctcgagacttttttccttgccgtatggccacttgttcccctcctacatccgccttccctgcaggcagactacgacgagttttgggaatggtgtcggaatagcgagaattctttaccttcggataaactccgcgatgacccaaccttgatctgcctgctctttgcagtcctatactgtggcgcatctgccgcgccggcagccagctgggtgaacaccaacctgcagggtctacagaaggagacgacagtgagccatctcaagtcggcatatacaacgagtctttctctatgtcaatatcaggaacatccaactttaaatacgctggtttcgaatttgctgacgggaccgtttctggatcggccgttcgagcccatgcgtagcctggttaacgtgagcaccacggtgcgcattgcccagactatggggttgcatcgggagggggtaggatctgcgctgagttccgttgatcgggaaatgcggcgccgggtctggtggcacattatttggcttgatgtgcagtcgagcatctccacggggctaaccccctgttgcgggaacgaggccttggatgcggtgggcatggttggcactgacaatgcagagccgagcgatatccccgctggaatttccccacccaacgaactggtgactaacagacagtcggtagccatgctatatgctatcgggcgcttccaggctgctcgcttacaggcgaggaccgtggcgcacctgcagagtgcgcatggtccaagccagcatggatttggcgagctgatcacagatgccaaggagcttctgcagaagattgactcgcttatcgcacgcgttccaacacaggggatccccgaaatgggatacataccatcccgcctggcgaatgcgtctccgtccacccagcccttgttgtacaaggacgattcaagccagccaaccgtctttgcagcatggacgcgaatcatgctaacattgctgaaatcagaaatggctatcttattgcagaaaccatttcttccacccccggacagtgcgaacccgcaatcgcgcaagtcatggaccagtatggcgcagctctgcgtgaattacttgcgtatttacctgcagctgtatcaggcccctgctttctccccatacgcgtggttctgctgcagccactatgggcctctccaatgcgtattcatcaccctaatgtacctccattacttccagcactctggagagaccacgctggcccgatattgtgttgatgaggttatacatcactgcgtcgcccagtatcaagctccaggaccttcctcgacaaggactagccttgatggtactgattccagtgggggcaaaatgccaatgccattggccattcaggttctcgttgaccttcacgaacggctcgactcgtctctcggacctgaagacaaggctccgccactggacctaatcgagtgtcaggcccgattttctatgtctcaccttgctaccaaggcgtccaacttgcgcgcgacgtctgaccagccttcttctgatgtatcgtctaccactcgcacccaccacaattgcgagacgcctcccataaccaccggggctcctccagttgctgctggtaacaaatcaatcccaccgaacagcgtctttgtggccgggagtgactcaggcttggatatggatttccttgctacgatttcggatcttgaggcctggtcctcgtcattgattctggaatccgacaatctcctcgcacgtcctgataatatgacgcctgatcatgctgtaatcacagggttaggctcacaaagtaccgcgactggtcgccgtggccttcccggcggactcgattttccagtgtaa

**Note S2** DNA sequence of dCas9-VPR fusion and description of its domains on vector pLM-AMA18.0-dCas9-VPR.

*atggacaagaagtattctatcggactggccatcgggactaatagcgtcgggtgggccgtgatcactgacgagtacaaggtgccctctaagaagttcaaggtgctcgggaacaccgaccggcattccatcaagaaaaatctgatcggagctctcctctttgattcaggggaaaccgctgaagcaacccgcctcaagcggactgctagacggcggtacaccaggaggaagaaccggatttgttaccttcaagagatattctccaacgaaatggcaaaggtcgacgacagcttcttccataggctggaagaatcattcctcgtggaagaggataagaagcatgaacggcatcccatcttcggtaatatcgtcgacgaggtggcctatcacgagaaatacccaaccatctaccatcttcgcaaaaagctggtggactcaaccgacaaggcagacctccggcttatctacctggccctggcccacatgatcaagttcagaggccacttcctgatcgagggcgacctcaatcctgacaatagcgatgtggataaactgttcatccagctggtgcagacttacaaccagctctttgaagagaaccccatcaatgcaagcggagtcgatgccaaggccattctgtcagcccggctgtcaaagagccgcagacttgagaatcttatcgctcagctgccgggtgaaaagaaaaatggactgttcgggaacctgattgctctttcacttgggctgactcccaatttcaagtctaatttcgacctggcagaggatgccaagctgcaactgtccaaggacacctatgatgacgatctcgacaacctcctggcccagatcggtgaccaatacgccgaccttttccttgctgctaagaatctttctgacgccatcctgctgtctgacattctccgcgtgaacactgaaatcaccaaggcccctctttcagcttcaatgattaagcggtatgatgagcaccaccaggacctgaccctgcttaaggcactcgtccggcagcagcttccggagaagtacaaggaaatcttctttgaccagtcaaagaatggatacgccggctacatcgacggaggtgcctcccaagaggaattttataagtttatcaaacctatccttgagaagatggacggcaccgaagagctcctcgtgaaactgaatcgggaggatctgctgcggaagcagcgcactttcgacaatgggagcattccccaccagatccatcttggggagcttcacgccatccttcggcgccaagaggacttctacccctttcttaaggacaacagggagaagattgagaaaattctcactttccgcatcccctactacgtgggacccctcgccagaggaaatagccggtttgcttggatgaccagaaagtcagaagaaactatcactccctggaacttcgaagaggtggtggacaagggagccagcgctcagtcattcatcgaacggatgactaacttcgataagaacctccccaatgagaaggtcctgccgaaacattccctgctctacgagtactttaccgtgtacaacgagctgaccaaggtgaaatatgtcaccgaagggatgaggaagcccgcattcctgtcaggcgaacaaaagaaggcaattgtggaccttctgttcaagaccaatagaaaggtgaccgtgaagcagctgaaggaggactatttcaagaaaattgaatgcttcgactctgtggagattagcggggtcgaagatcggttcaacgcaagcctgggtacttaccatgatctgcttaagatcatcaaggacaaggattttctggacaatgaggagaacgaggacatccttgaggacattgtcctgactctcactctgttcgaggaccgggaaatgatcgaggagaggcttaagacctacgcccatctgttcgacgataaagtgatgaagcaacttaaacggagaagatataccggatggggacgccttagccgcaaactcatcaacggaatccgggacaaacagagcggaaagaccattcttgatttccttaagagcgacggattcgctaatcgcaacttcatgcaacttatccatgatgattccctgacctttaaggaggacatccagaaggcccaagtgtctggacaaggtgactcactgcacgagcatatcgcaaatctggctggttcacccgctattaagaagggtattctccagaccgtgaaagtcgtggacgagctggtcaaggtgatgggtcgccataaaccagagaacattgtcatcgagatggccagggaaaaccagactacccagaagggacagaagaacagcagggagcggatgaaaagaattgaggaagggattaaggagctcgggtcacagatccttaaagagcacccggtggaaaacacccagcttcagaatgagaagctctatctgtactaccttcaaaatggacgcgatatgtatgtggaccaagagcttgatatcaacaggctctcagactacgacgtggctgccatcgtccctcagagcttcctcaaagacgactcaattgacaataaggtgctgactcgctcagacaaggcccggggaaagtcagataacgtgccctcagaggaagtcgtgaaaaagatgaagaactattggcgccagcttctgaacgcaaagctgatcactcagcggaagttcgacaatctcactaaggctgagaggggcggactgagcgaactggacaaagcaggattcattaaacggcaacttgtggagactcggcagattactaaacatgtcgcccaaatccttgactcacgcatgaataccaagtacgacgaaaacgacaaacttatccgcgaggtgaaggtgattaccctgaagtccaagctggtcagcgatttcagaaaggactttcaattctacaaagtgcgggagatcaataactatcatcatgctcatgacgcatatctgaatgccgtggtgggaaccgccctgatcaagaagtacccaaagctggaaagcgagttcgtgtacggagactacaaggtctacgacgtgcgcaagatgattgccaaatctgagcaggagatcggaaaggccaccgcaaagtacttcttctacagcaacatcatgaatttcttcaagaccgaaatcacccttgcaaacggtgagatccggaagaggccgctcatcgagactaatggggagactggcgaaatcgtgtgggacaagggcagagatttcgctaccgtgcgcaaagtgctttctatgcctcaagtgaacatcgtgaagaaaaccgaggtgcaaaccggaggcttttctaaggaatcaatcctccccaagcgcaactccgacaagctcattgcaaggaagaaggattgggaccctaagaagtacggcggattcgattcaccaactgtggcttattctgtcctggtcgtggctaaggtggaaaaaggaaagtctaagaagctcaagagcgtgaaggaactgctgggtatcaccattatggagcgcagctccttcgagaagaacccaattgactttctcgaagccaaaggttacaaggaagtcaagaaggaccttatcatcaagctcccaaagtatagcctgttcgaactggagaatgggcggaagcggatgctcgcctccgctggcgaacttcagaagggtaatgagctggctctcccctccaagtacgtgaatttcctctaccttgcaagccattacgagaagctgaaggggagccccgaggacaacgagcaaaagcaactgtttgtggagcagcataagcattatctggacgagatcattgagcagatttccgagttttctaaacgcgtcattctcgctgatgccaacctcgataaagtccttagcgcatacaataagcacagagacaaaccaattcgggagcaggctgagaatatcatccacctgttcaccctcaccaatcttggtgcccctgccgcattcaagtacttcgacaccaccatcgaccggaaacgctatacctccaccaaagaagtgctggacgccaccctcatccaccagagcatcaccggactttacgaaactcggattgacctctcacagctcggaggg*gatagcagggctgac**cccaagaagaagaggaaggtg**gaggccagcggttccggacgggctgacgcattggacgattttgatctggatatgctgggaagtgacgccctcgatgattttgaccttgacatgcttggttcggatgcccttgatgactttgacctcgacatgctcggcagtgacgcccttgatgatttcgacctggacatgctgattaactctagaagttccggatct**ccgaaaaagaaacgcaaagtt**ggtagccagtacctgcccgacaccgacgaccggcaccggatcgaggaaaagcggaagcggacctacgagacattcaagagcatcatgaagaagtcccccttcagcggccccaccgaccctagacctccacctagaagaatcgccgtgcccagcagatccagcgccagcgtgccaaaacctgccccccagccttaccccttcaccagcagcctgagcaccatcaactacgacgagttccctaccatggtgttccccagcggccagatctctcaggcctctgctctggctccagcccctcctcaggtgctgcctcaggctcctgctcctgcaccagctccagccatggtgtctgcactggctcaggcaccagcacccgtgcctgtgctggctcctggacctccacaggctgtggctccaccagcccctaaacctacacaggccggcgagggcacactgtctgaagctctgctgcagctgcagttcgacgacgaggatctgggagccctgctgggaaacagcaccgatcctgccgtgttcaccgacctggccagcgtggacaacagcgagttccagcagctgctgaaccagggcatccctgtggcccctcacaccaccgagcccatgctgatggaataccccgaggccatcacccggctcgtgacaggcgctcagaggcctcctgatccagctcctgcccctctgggagcaccaggcctgcctaatggactgctgtctggcgacgaggacttcagctctatcgccgacatggacttctccgcactgctgggtagcggatcgggatctcgggattccagggaagggatgtttttgccgaagcctgaggccggctccgctattagtgacgtgtttgagggccgcgaggtgtgccagccaaaacgaatccggccatttcatcctccaggaagtccatgggccaaccgcccactccccgccagcctcgcaccaacaccaaccggtccagtacatgagccagtcgggtcactgaccccggcaccagtccctcagccactggatccagcgcccgcagtgactcccgaggccagtcacctgttggaggatcccgatgaagagacgagccaggctgtcaaagcccttcgggagatggccgatactgtgattccccagaaggaagaggctgcaatctgtggccaaatggacctttcccatccgcccccaaggggccatctggatgagctgacaaccacacttgagtccatgaccgaggatctgaacctggactcacccctgaccccggaattgaacgagattctggataccttcctgaacgacgagtgcctcttgcatgccatgcatatcagcacaggactgtccatcttcgacacatctctgttttga
*dCas9m4: green italics*, **SV40-NLS: red bold**, VP64: yellow underlined, (RelA) AD: purple, Rta AD: blue underlined

**References**

1. Pohl, C., Mózsik, L., Driessen, A. J. M., Bovenberg, R. A. L. & Nygård, Y. I. Genome Editing in Penicillium chrysogenum Using Cas9 Ribonucleoprotein Particles. in *Synthetic Biology. Methods in Molecular Biology* (ed. ed.Braman J.) vol. 1772 213–232 (Humana Press Inc., 2018).

2. Mózsik, L., Büttel, Z., Bovenberg, R. A. L., Driessen, A. J. M. & Nygård, Y. Synthetic control devices for gene regulation in Penicillium chrysogenum. *Microb. Cell Fact.* **18**, (2019).

3. Bovenberg R.A.L., Kiel, J. A. K. W., Wenzel, T. J., Los, A. P. Vector-Host system. *World Intellect. Prop. Organ.* Application number WO/2012/123429 (2012).

4. Pohl, C. *et al.* A Penicillium rubens platform strain for secondary metabolite production. *Sci. Rep.* **10**, 7630 (2020).

5. Lee, M. E., DeLoache, W. C., Cervantes, B. & Dueber, J. E. A Highly Characterized Yeast Toolkit for Modular, Multipart Assembly. *ACS Synth. Biol.* **4**, 975–986 (2015).

6. Chavez, A. *et al.* Highly efficient Cas9-mediated transcriptional programming. *Nat. Methods* **12**, 326–328 (2015).

7. Nødvig, C. S., Nielsen, J. B., Kogle, M. E. & Mortensen, U. H. A CRISPR-Cas9 system for genetic engineering of filamentous fungi. *PLoS One* **10**, (2015).

8. Weber, E., Engler, C., Gruetzner, R., Werner, S. & Marillonnet, S. A modular cloning system for standardized assembly of multigene constructs. *PLoS One* **6**, (2011).

9. Pohl, C., Kiel, J. A. K. W., Driessen, A. J. M., Bovenberg, R. A. L. & Nygård, Y. CRISPR/Cas9 Based Genome Editing of Penicillium chrysogenum. *ACS Synth. Biol.* **5**, 754–764 (2016).
